# An optimised tissue disaggregation and data processing pipeline for characterising fibroblast phenotypes using single-cell RNA sequencing

**DOI:** 10.1038/s41598-019-45842-4

**Published:** 2019-07-03

**Authors:** Sara Waise, Rachel Parker, Matthew J. J. Rose-Zerilli, David M. Layfield, Oliver Wood, Jonathan West, Christian H. Ottensmeier, Gareth J. Thomas, Christopher J. Hanley

**Affiliations:** 1 Cancer Sciences Unit, University of Southampton, Southampton, UK; 2 Institute for Life Sciences, University of Southampton, Southampton, UK; 3 Cancer Research UK and NIHR Southampton Experimental Cancer Medicine Centre, Southampton, UK

**Keywords:** Isolation, separation and purification, Bioinformatics

## Abstract

Single-cell RNA sequencing (scRNA-Seq) provides a valuable platform for characterising multicellular ecosystems. Fibroblasts are a heterogeneous cell type involved in many physiological and pathological processes, but remain poorly-characterised. Analysis of fibroblasts is challenging: these cells are difficult to isolate from tissues, and are therefore commonly under-represented in scRNA-seq datasets. Here, we describe an optimised approach for fibroblast isolation from human lung tissues. We demonstrate the potential for this procedure in characterising stromal cell phenotypes using scRNA-Seq, analyse the effect of tissue disaggregation on gene expression, and optimise data processing to improve clustering quality. We also assess the impact of *in vitro* culture conditions on stromal cell gene expression and proliferation, showing that altering these conditions can skew phenotypes.

## Introduction

In recent years, a number of platforms to perform single cell RNA sequencing (scRNA-seq) have been developed. These technologies facilitate analysis of biological processes at a previously unachievable resolution, providing novel insights into the multicellular ecosystems present in normal tissues and pathogenic processes^1–4^. However, to harness the full potential of this approach, a number of technical obstacles must be overcome.

The first consideration for scRNA-seq is the approach used to generate the single-cell suspension for analysis. Suspensions generated using mechanical and/or enzymatic protocols may not accurately mirror the tissue of origin. Lengthy or over-vigorous disaggregation may alter gene expression in cells^5^, confounding downstream analysis. In addition, some populations (*e*.*g*. epithelial and stromal cells) are often relatively under-represented in scRNA-seq^3^. Fibroblasts, almost ubiquitous in human tissues, are embedded within extracellular matrix and are particularly difficult to isolate. This heterogeneous cell population^6–15^ plays a major role in tissue homeostasis and disease pathogenesis, including cancer progression^16–19^. However, fibroblasts remain a poorly-defined group: commonly-used fibroblast markers (α-smooth muscle actin, α-SMA; platelet-derived growth factor, PDGFR; and fibroblast activation protein-α, FAP-α) are also expressed on other cell types^20,21^. While fibroblasts are relatively straightforward to culture *ex vivo*, it is unclear whether standard approaches^22,23^ retain the distinct fibroblast phenotypes found *in vivo*^6–15^. In order to accurately characterise fibroblast subtypes and investigate functional differences, precise phenotyping at a single-cell resolution is required.

Filtering out low-quality events (such as sequenced cell fragments, or cells sequenced in insufficient depth) is an essential quality-control step prior to downstream analysis of scRNA-seq data. The approach used to perform filtering is not standardised and varies considerably between studies, potentially compromising reproducibility. Variability can be attributable to inherent differences in data generated by different platforms. For example, due to the greater sequencing depth afforded by SMART-seq2^24^, the lowest acceptable number of genes per cell (nGene) for datasets generated using this platform will be considerably higher than for data generated with droplet-based technologies (*e*.*g*. 10X and Drop-Seq)^1,3^. The relative affordability and commercial availability of droplet-based platforms have resulted in these approaches becoming increasingly popular. However, their relatively low sequencing depths means that identification of low-quality cells can be challenging, particularly when analysing mixed cell type populations.

Here, we compare previously-described approaches for tissue disaggregation. We determine the optimal method for stromal cell isolation from primary human lung tissue (allowing immediate analysis without *in vitro* culture) and refine the single-cell bioinformatic analysis pipeline for our data. We also examine how *in vitro* culture alters the transcriptomes of isolated primary fibroblasts, and assess the impact of altering culture conditions on fibroblast phenotype.

## Results

### Disaggregation enzymes and incubation times have a significant impact on stromal cell isolation

Flow cytometry was used to determine the fraction of fibroblasts isolated from lung tissues using different enzyme cocktails. To optimise fibroblast identification, we excluded immune (CD45+), epithelial (EpCAM+) and endothelial (CD31+) cells, and compared the expression of three previously-described fibroblast surface markers (PDGFR-α and -β, CD90) in foetal lung (IMR-90) and skin (HFFF2) fibroblasts and also a lung cancer cell line (H441). CD90 was found to be a robust marker of lung fibroblasts with greater sensitivity and specificity than PDGFR-α alone, or in combination with PDGFR-β, and was therefore used in all further analyses (CD90: 99.2% positivity with 1175-fold increase in mean fluorescence intensity compared to the negative control; PDGFR-α: 4.1% positivity with 4.6-fold increase in mean fluorescent intensity (MFI); and PDGFR-α with –β: 9.2% positivity with 19-fold increase in MFI; Fig. S1).

**Figure 1:**
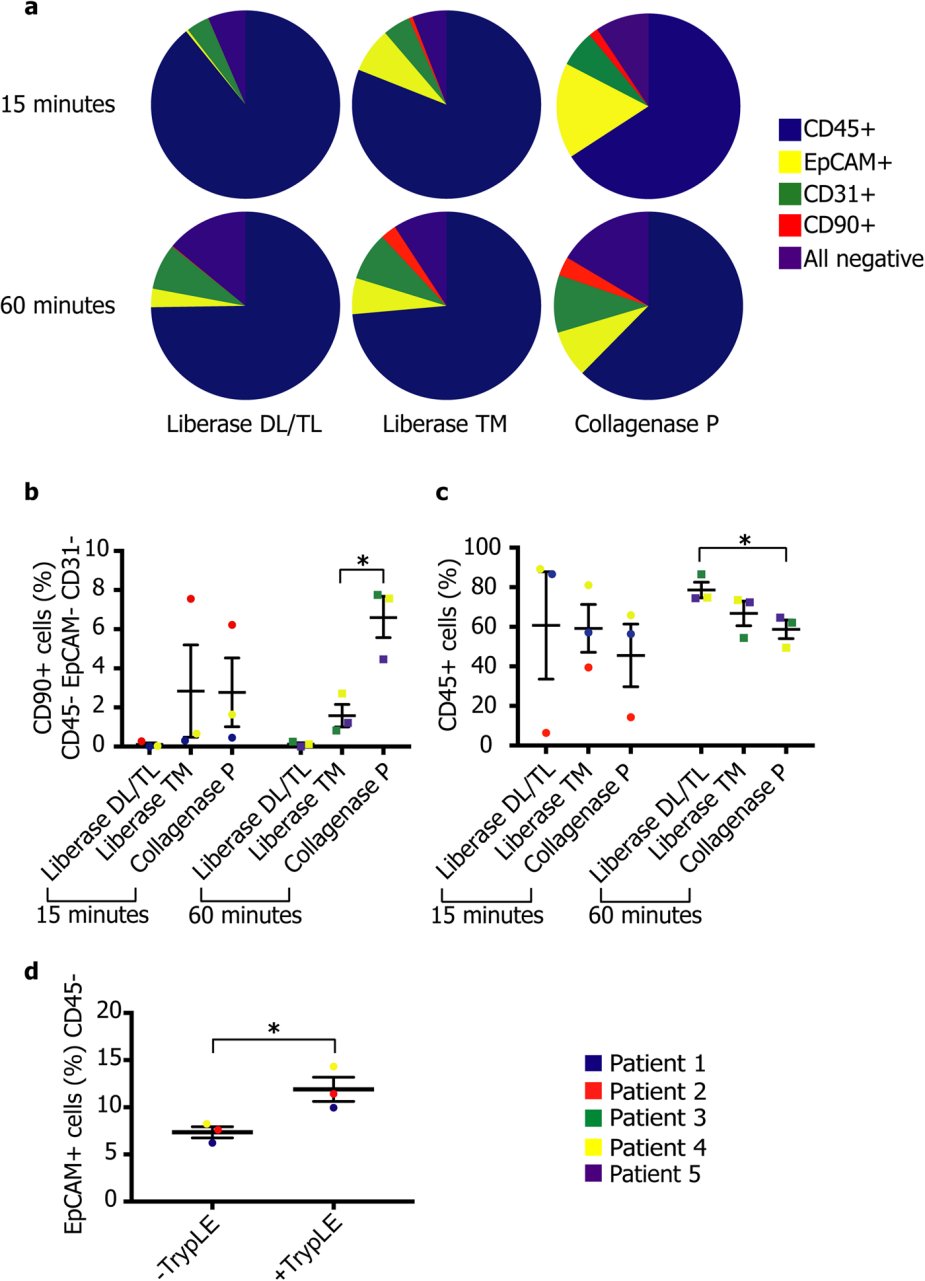
Disaggregation enzymes and incubation times have a significant impact on stromal cell isolation. (**a**) Representative pie charts for each disaggregation time and enzyme cocktail. (**b**–**d**) Dot plots showing cell-type fractions isolated by different disaggregation procedures across human patient samples (n = 5): fibroblasts (CD45-EpCAM-CD31-CD90+: **b**), Immune cells (CD45+: **c**), and epithelial cells (CD45-EpCAM+: **d**; *p < 0.05, unpaired two-tailed *t*-test). Further data associated with this figure can be found in Figs S1–4.

We next examined the effect of disaggregation time and different protease cocktails (Collagenase P, Liberase DL, TL and TM) on fibroblast isolation from lung tissues. Shorter disaggregation times (15 minutes) and lower protease-strength enzymes (Liberase DL, TL and TM) were insufficient to isolate stromal cells; instead yielding high proportions of immune cells (CD45+). In contrast, tissue digestion with Collagenase P for 60 minutes resulted in a greater diversity of cell types isolated (Fig. 1a), with a significantly lower proportion (*p = *0.03) of CD45+ cells and a significantly higher proportion (*p = *0.01) of CD45-EpCAM-CD31-CD90+ cells (*i*.*e*. fibroblasts; Fig. 1b,c). There was no significant change in the fractions of CD45-EpCAM+ (epithelial) or CD45-EpCAM-CD31+ (endothelial) cells (Fig. S2a,b). Dissociation enzyme and duration did not significantly affect cell yield or viability (Fig. S2c,d).

Unexpectedly, analysis also revealed that epithelial cells represented a low proportion of the cells isolated. We hypothesised that this was due to tight intercellular interactions between these cells, with resulting cell aggregates removed during filtration or at FACS analysis. To test this, following Collagenase P incubation for 60 minutes, single cell suspensions were additionally incubated with TrypLE (Thermo Fisher) at 37 °C for 10 minutes. This yielded a significantly higher proportion of epithelial cells (Fig. 1d), accompanied by a reduced immune cell fraction (Fig. S3a). Percentages of endothelial cells and fibroblasts were not significantly affected (Fig. S3b,c).

### Lung tissue disaggregation with Collagenase P for sixty minutes is compatible with single-cell RNA sequencing using the Drop-seq platform

Development of scRNA-Seq has facilitated identification of novel mesenchymal sub-populations in, for example, murine lung^4^. To determine the compatibility of our optimised disaggregation protocol with this approach to gene expression profiling, we processed primary human tissue (from a lung affected by granulomatous inflammation) and captured single-cell transcriptomes using Drop-seq^25^. Quantification and fragment analysis of PCR-amplified cDNA (Fig. S4) in these samples confirmed the successful capture of full-length transcripts (up to 5 Kb, suggesting that mRNA integrity was unaffected).

### Identifying and removing low-quality droplets

**Figure 2:**
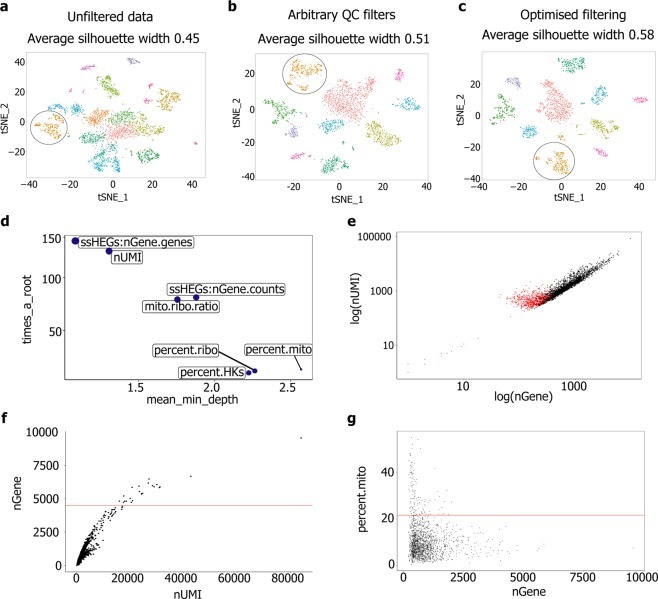
Standardised quality-control metrics improve clustering quality of scRNA-seq data. tSNE plots showing principle component-based clustering and average silhouette width for: (**a**) unfiltered data, (**b**) data filtered using widely-used quality-control metrics and (**c**) the optimised approach described here (shown in **d**–**g**). Each point represents an individual cell, groups of cells with similar transcriptomes are referred to as a ‘cluster’ and distinguished by colour. Fibroblast populations are encircled in black. (**d**–**g**) Cell filtering using our optimised processing pipeline: (**d**) Multi-way importance plot showing the relative importance of each metric in distinguishing between low-quality events and droplets, (**e**) Plot of log(nGene) *vs*. log(nUMI). Data points identified as low-quality events are highlighted in red, (**f**) Plot of nUMI *vs*. nGene. The red line indicates the upper nGene threshold (defined as 2.5 MAD above the median) and (**g**) Plot of nGene *vs*. percent mito. The red line indicates the upper threshold (defined as 2.5 MAD above the median). Further data associated with this figure can be found in Fig. S5.

There are currently no standardised approaches for this pre-processing step, and filtering thresholds vary between studies^1,3^. We therefore sought to develop a generalisable approach to pre-processing droplet-based scRNA-Seq data, in order to improve filtering and thus cell type identification by unsupervised clustering. Baseline clustering quality was assessed using unfiltered data. This identified 17 clusters, with an average silhouette width of 0.45 (Fig. 2a). We also applied previously-described pre-processing steps (removing cells with more than 15000 or fewer than 200 nUMIs, over 5000 or below 100 genes, or over 20% of reads mapping to mitochondrial genes)^1–3^, finding that this improved clustering quality (average silhouette width 0.51; Fig. 2b).

Due to the lower read depth associated with droplet-based sequencing platforms, distinguishing low-quality droplets from true cells with a low nGene (*e*.*g*. lymphocytes^3^) is challenging. This is further complicated by the absence of ‘ground truth’ labels for low- and high-quality events for these technologies. To determine the optimal approach, we examined the variation of a range of QC metrics between events assumed to be low-quality (nGene less than 100) and those likely to represent true cells (nGene between the median and 2.5 MAD above the median; Fig. S5a). QC metrics comprised: nUMI, the percentage of reads mapping to mitochondrial (percent mito) or ribosomal genes (percent ribo), the ratio between these two values, and housekeeping gene expression. We additionally defined an algorithm to estimate the contribution of both reads and genes detected due to encapsulation of ‘ambient’ RNA (described in the Materials and methods section). Each of these metrics showed a significant difference between the low-quality droplets and true cells (Bonferroni corrected *p* < 0.0001 Fig. S5b–h).

To harness the predictive power of each variable, we trained a machine learning (random forest^26^) model to distinguish between the low-quality droplet and true cell groups. The data was divided at random into ‘training’ and ‘test’ datasets (in a ratio of 3 to 1). When applying the trained classifier to the ‘test’ dataset, ‘cells’ were detected at a sensitivity and specificity of 100%. Analysing the relative importance of each metric in the classifier^27^ showed that the ratio between the number of the sample specific highly expressed genes detected and total number of genes detected (ssHEGs:nGene.genes) was the most important variable in distinguishing between cells and low-quality droplets (Fig. 2d). Highlighting the droplets identified as low-quality by the classifier, on a log(nUMI) *vs*. log(nGene) plot (Fig. 2e), illustrates that this machine learning approach to filtering provides greater sensitivity than filtering using hard thresholds for individual QC metrics. Cell-cell doublets were identified on an nUMI *vs*. nGene plot (Fig. S2f) and removed from downstream analysis. Dead or dying cells can also impact downstream analysis; percent mito is known to be an effective measure of this process in scRNA-Seq data^28^. We removed cells with a percent mito greater than 2.5 MAD above the median (Fig. 2g). This combined approach further improved the quality of clustering (average silhouette width 0.58; Fig. 2c).

### Assessing the impact of extended enzymatic disaggregation times on transcriptomic data

**Figure 3:**
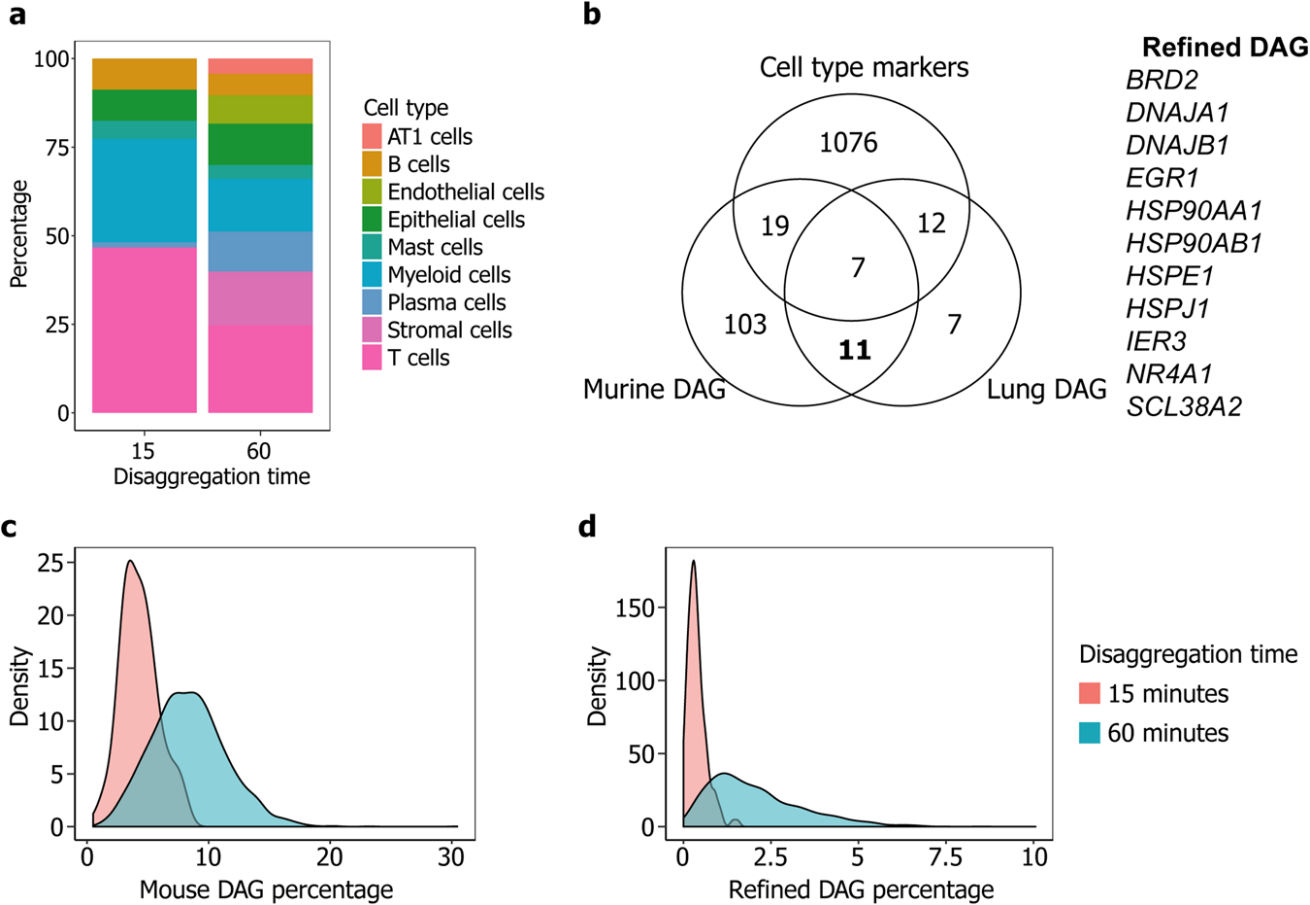
Longer tissue disaggregation enables detection of more cell types, with concomitant increases in disaggregation-associated changes in gene expression. (**a**) Stacked barplot showing cell type fractions generated by disaggregation for 15 and 60 minutes. (**b**) Venn diagram showing overlap between murine disaggregation-associated signature^5^ (Murine DAG), human disaggregation-associated signature (Lung DAG) and cell type markers. The 11 genes comprising the refined signature (highlighted in bold) are shown on the right. (**c**) and (**d**) Density plots showing percentage expression of the murine disaggregation signature (**c**) and the refined disaggregation signature (**d**), in human lung tissue disaggregated for 15 and 60 minutes. Further data associated with this figure can be found in Fig. S6.

Analysis of the scRNA-seq data confirmed that disaggregation for 60 minutes yields a greater fraction of stromal cells (as well as more even coverage of other cell types) than disaggregation for 15 minutes (Fig. 3a). However, previous authors have shown that enzymatic disaggregation can lead to changes in gene expression, describing a disaggregation-associated gene signature (derived using murine stem cells)^5^. We assessed the effect of applying this signature to our data: as expected, its expression was increased in samples disaggregated for 60 minutes compared to those processed for 15 minutes (Fig. 3c). However, we found that this signature did not appear to impact cell clustering, and was not a prominent feature of any individual cluster (Fig. S6a).

As the gene signature proposed by van den Brink *et al*. was derived from murine experiments and includes previously-described markers for human cell types, we sought to refine this signature based on our analysis. We therefore cross-referenced the genes in this signature with those upregulated (*p* < 0.001, average log_10_(fold change) > 1) following disaggregation for 60 minutes *vs*. 15 minutes, excluding those identified as cell type markers. This identified a list of eleven genes which were consistently upregulated following extended disaggregation, and were not upregulated by a particular cell type (Fig. 3b). We applied this list to our data as a refined disaggregation signature.

The distribution of expression of this signature in our dataset is shown in Figs 3d and S6b. Clusters 0, 4 and 9 (representing T cells, B cells and mast cells) show higher expression, indicating that some cell types may be more sensitive to extended enzymatic disaggregation than others. However, none of the principle components used for clustering correlated with this gene signature (ρ < 0.23). It is therefore unlikely that disaggregation-associated gene changes have impacted cell clustering. Removal of cells with high expression (greater than 2.5 MAD above the median) of this signature did not improve clustering quality (maximum average silhouette width 0.56 *vs*. 0.58 prior to filtering). However, the potential impact of extended enzymatic disaggregation on cellular transcriptomes should be considered when analysing such data: in particular, where particular cell populations appear to be defined by high expression of a disaggregation-associated signature.

### *In vitro* culture alters transcriptomes

**Figure 4:**
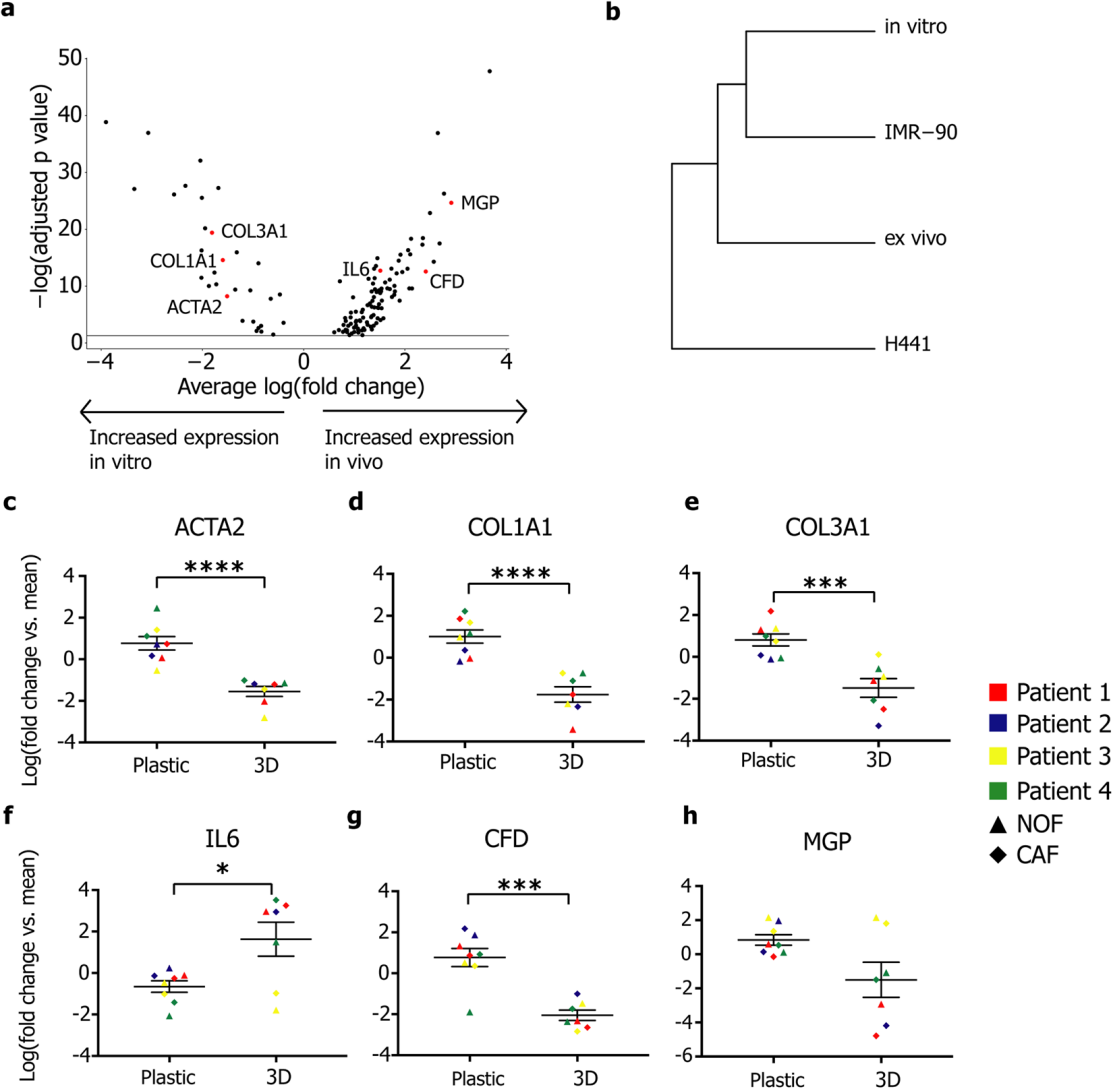
*In vitro* culture alters fibroblast transcriptomes. (**a**) Volcano plot of genes differentially expressed (log(fold change) >1) between *in vitro* and *ex vivo* fibroblasts. Genes selected for analysis at real time-PCR are highlighted in red. (**b**) Dendrogram showing unsupervised hierarchical clustering of average gene expression for the four cell populations. (**c**–**g**) Dot plots showing changes in gene expression (real-time PCR) in primary lung fibroblasts following culture on plastic or in 3D (collagen:Matrigel gels): (**c**) *ACTA2*, (**d**) *COL1A1*, (**e**) *COL3A1*, (**f**) *IL6*, (**g**) *CFD* and (**h**) *MGP*. Gene expression levels expressed as the log2 fold change relative to mean expression across all samples (n = 4). ▲: NOF, ♦: CAF. *p < 0.05, ***p < 0.001, ****p < 0.0001, unpaired or Welch’s *t*-tests. Further data associated with this figure can be found in Figs S7–S9.

Recent studies have shown that the gene expression profiles of cancer-associated fibroblasts are significantly altered by culture *in vitro*^1,2^. We cultured primary fibroblasts isolated from tumour and non-involved tissue *in vitro* for one passage and compared gene expression profiles to those of fibroblasts analysed immediately following disaggregation (“*ex vivo*”), and a lung foetal fibroblast cell line (IMR-90). We found that fibroblasts isolated from tumour or non-involved tissues show differential gene expression following culture *in vitro* for one passage (Fig. 4a). Gene expression by these cells more closely resembles IMR-90 cells than *ex vivo* fibroblasts (Fig. 4b), showing that culture on plastic surfaces causes significant transcriptomic changes in primary lung fibroblasts.

### 3D *in vitro* culture significantly alters gene expression and can be used to skew fibroblast phenotypes towards previously described sub-types

To determine whether *ex vivo* fibroblast phenotypes can be recapitulated or retained by manipulation of *in vitro* conditions, we cultured primary fibroblasts either on plastic or in collagen-Matrigel® 3D gels. Cells were harvested after five days and changes in gene expression measured using real-time PCR.

Alteration of culture conditions had a significant impact on the expression of genes associated with previously-described fibroblast subtypes (Fig. 4c–f; these genes were also found to be differentially expressed between *ex vivo* and *in vitro* fibroblasts; Fig. 4a and Table 1). Expression of *ACTA2*, *COL1A1* and *COL3A1* (markers of myofibroblast transdifferentiation^29,30^) were significantly increased in fibroblasts grown in 2D, whereas expression of *IL6* (a marker of ‘inflammatory’ fibroblasts^31^) was significantly increased in 3D. However, 3D culture conditions were not sufficient to recover *ex vivo* fibroblast gene expression: *CFD* and *MGP* (genes which were upregulated in *ex vivo* fibroblasts) showed significant downregulation and a non-significant reduction in expression, respectively (Fig. 4f,g). None of the investigated genes showed significant differential expression between fibroblasts from normal (NOF) or tumour (CAF) samples or across patients (Fig. S8). The changes in expression of COL1A1, SMA and IL6 were found to be maintained at the protein level (Fig. S9).

**Table 1:** Summary of key genes differentially expressed between *ex vivo* and *in vitro* fibroblasts.

Gene	Average log(fold change)	Adjusted *p* value
*ACTA2*	−1.51	5.93 × 10^−9^
*COL1A1*	−1.60	2.60 × 10^−15^
*COL3A1*	−1.81	3.94 × 10^−20^
*IL6*	1.51	1.88 × 10^−13^
*CFD*	2.41	2.65 × 10^−13^
*MGP*	2.92	2.20 × 10^−25^

## Discussion

Fibroblasts are a heterogeneous population in both normal and disease states^16,30,32,33^. Here, we describe a protocol for isolation of stromal cells by disaggregation of primary human lung tissues, enabling these cells to be analysed directly *ex vivo*. In addition, this protocol may be modified to isolate other cell types: addition of TrypLE generates a significantly higher fraction of epithelial cells without affecting the proportions of stromal cells. We also examine the effect of tissue disaggregation on gene expression, and optimise pre-processing of scRNA-Seq data. Having applied these approaches, we additionally demonstrate that culturing fibroblasts *in vitro* significantly impacts molecular phenotype: this has important consequences for functional analysis of these cells from normal and pathological tissues.

In keeping with recent findings^3^, our results show that extended Collagenase incubation times are required to release fibroblasts from tissue samples. In contrast, non-adherent cells (such as immune cells) are readily and rapidly isolated by enzymatic disaggregation. Although prolonged incubation with bacterial Collagenases has been reported to cause cellular damage through proteolysis^34^, we did not observe any significant differences in cell viability between the conditions investigated here. The described protocol is therefore likely to be a superior method for cell isolation compared with mechanical disaggregation alone: although mechanical disaggregation preserves surfaces markers, this approach has been reported to give lower cell yield and viability^35,36^.

Previous authors have described a disaggregation-associated gene signature, based on the changes in gene expression induced by prolonged enzymatic disaggregation^5^. As this signature was derived from murine muscle stem cells, we sought to determine its’ applicability to human lung tissues. We found eleven genes from this signature that were both upregulated in cells isolated from human lung tissues following prolonged disaggregation, and not identified as cell type markers. As these genes were conserved across both species and tissues, we suggest that this refined gene list represents a robust disaggregation-associated signature. We propose that this signature will assist in distinguishing true clusters from those arising due to disaggregation-induced changes in gene expression. Accounting for this phenomenon is likely to be critical when attempting to identify cell subpopulations. This signature may be used either to filter out all cells with high expression, or to exclude principle components which correlate with disaggregation-associated gene expression changes from downstream cell clustering.

Enzymatic disaggregation can impact surface marker expression through indiscriminate protease activity^35–37^. This may limit its use for certain downstream assays (*e*.*g*. immunophenotyping by flow cytometry) if the markers of interest are susceptible to proteolytic cleavage by the Collagenase P enzyme cocktail. However, changes in the expression of surface proteins are not reflected by a corresponding change in mRNA, and are unlikely to significantly impact single-cell RNA sequencing or longer-term tissue culture. For example, expression of *CDH1* (the gene encoding E-cadherin, a surface protein susceptible to protease cleavage^38^) is detectable in our epithelial cell populations.

Regardless of the platform used, scRNA-seq data requires quality-control measures to remove low-quality events. There are currently no standardised approaches for this pre-processing step, and filtering thresholds vary between studies^1,3^. Routinely-applied quality control metrics used for filtering low-quality events include exclusion of cells with a low number of genes sequenced (nGene; removing sequenced cell fragments) or a high percentage of reads mapping to mitochondrial genes (‘percent mito’, indicative of burst cells that have lost cytoplasmic contents and dead or dying cells). Single-cell platforms are also known to be affected by cell doublets: where libraries are sequenced from co-isolated, rather than single, cells^24,25^. These events are easily identified as outliers for nGene on a plot of nUMI *vs*. nGene.

We developed a standardised, generalisable method using a machine learning (random forest) approach to identify low-quality droplets based on a number of metrics. This enables removal of low-quality events from further analysis and improved clustering quality when compared with the most commonly-used current approaches.

A number of studies have demonstrated that mechanical changes to tissue culture substrates can impact fibroblast phenotypes^39–42^, yet the vast majority of research analysing these cells is still performed on plastic. We found that culturing fibroblasts *in vitro* causes a significant shift in phenotype compared to that seen immediately following isolation, consistent with previous studies^1,2^. This emphasises the importance of profiling cells directly from tissues to characterise accurately *in vivo* phenotypes.

Notably, we also show that fibroblasts show differential expression of genes associated with previously-described phenotypes when cultured in 2D or 3D. For example, primary human fibroblasts cultured on plastic surfaces showed increased expression of *COL1A1* and *COL3A1*, which are associated with a ‘myofibroblastic’ phenotype. In contrast, culturing these cells in 3D resulted in significantly increased expression of *IL6*, which may be indicative of an ‘inflammatory’ CAF phenotype^31^. The skewing of phenotypes induced by culture conditions needs to be taken into account when drawing conclusions from *in vitro* studies of fibroblast function.

Recent technological advances, such as Drop-seq, have enabled precise transcriptomic analysis of tissues at a single-cell level. Here, we describe a robust method for the isolation of fibroblasts. The protocols described address some of the challenges associated with isolating and analysing fibroblasts from lung tissue. These results will help to inform future studies into the phenotype and function of this poorly-defined cell type.

## Methods

### Human samples

Lung samples were received fresh from patients undergoing surgery at Southampton General Hospital (TargetLung study; approved by NRES Committee South Central: Hampshire A, REC number 14/SC/0186. All research was performed in accordance with the appropriate regulations. Informed consent was obtained from patients or their legal guardians). Samples were transported in 5 ml serum-free Dulbecco’s Modified Eagle Medium (DMEM; Sigma-Aldrich) on ice directly (within 1 hour) to the laboratory for tissue disaggregation.

### Tissue disaggregation

Tissue samples were washed in phosphate-buffered saline (PBS) to remove excess blood, then incubated for five minutes in PBS with Amphotericin B (250 pg/ml; Gibco). Samples were incised 10–15 times to relax the tissue and added to 5 ml of ‘complete’ DMEM (supplemented with L-glutamine (1% v/v; Sigma), foetal calf serum (10% v/v; Biosera) and penicillin-streptomycin (1% v/v; Sigma)). DNase (0.4 U/ml; Sigma) and the appropriate enzymatic mixture (Collagenase P, 3 U/ml; Sigma, Liberase DL, 0.25 U/ml; Sigma, Liberase TL, 1 U/ml; Roche, or Liberase TM, 0.25 U/ml; Sigma) were then added and samples were incubated at 37 °C with agitation (200 rpm) for 15 or 60 minutes.

To disrupt larger pieces of tissue during the incubation period, sequential pipetting (using 25 ml, 10 ml and 5 ml pipettes) was performed at 15, 30 and 60 minutes. The resulting suspension was strained through a 40 μm-pore filter (Corning), washed with 10 ml serum-free DMEM, and centrifuged at 450 *g* for 5 minutes. The pellet was re-suspended in 2 ml red cell lysis buffer (BioLegend) and incubated at 4 °C for 10 minutes. ‘Complete’ DMEM (10 ml) was added, and the samples centrifuged at 450 *g* for 5 minutes. The resulting pellet was re-suspended in 1 ml of either cell suspension buffer (90% ddH_2_O; 9% Optiprep, Sigma; 1% PBS with 0.1% bovine serum albumin; BSA) for use in Drop-Seq, or fluorescence-activated cell sorting (FACS) buffer (PBS with 0.5% w/v BSA and 0.05% w/v sodium azide) for analysis by flow cytometry.

### Flow cytometry

Flow cytometry was used to determine the relative proportions of cell types in single-cell suspensions. Cells were re-suspended in FACS buffer at a concentration of 1 × 10^6^/ml and stained with PE anti-CD31 (5 μg/μl; WM59), FITC anti-CD45 (10 μg/μl; H130), APC anti-CD90 (10 μg/μl; RPA-T4), Pacific Blue anti-EpCAM (5 μg/μl; 9C4), and 7-AAD (200 μg/μl) (BioLegend). Stained cells were analysed using a FACS Canto II (BD Biosciences). For all analyses, gating was performed as follows: 7-AAD *vs*. forward scatter area for live cells, forward scatter area *vs*. forward scatter height to exclude cell doublets, and side scatter area *vs*. forward scatter area to exclude debris. Gating to identify populations positive for each antibody was performed using forward scatter area *vs*. the relevant fluorescent channel in comparison to fluorescence minus one controls. All data were analysed using the FlowJo software package (version 10.2; FlowJo, LLC).

### Cell culture

Primary fibroblasts were isolated from the single-cell suspension generated following tissue disaggregation: the suspension was plated to the largest possible surface area at 100,000 cells/cm^2^ and incubated at 37 °C for 2 hours to allow cells to adhere. Following this, cells were washed in PBS (x3) to remove non-adherent (predominantly immune) cells. Adherent cells were then cultured in ‘Complete’ DMEM in a humidified incubator at 37 °C and 5% CO_2_. Details of tissue culture substrate coating experiments are given in the supporting information.

### RNA extraction

Cell pellets (from gel disaggregation or cell trypsinisation) underwent RNA extraction with DNase digestion using Reliaprep^TM^ RNA Cell Miniprep System (Promega). RNA quantitation was performed using the NanoDrop Spectrophotometer (Thermo Fisher Scientific).

### cDNA synthesis and quantitative real-time PCR

One microgram of RNA was reverse transcribed in a 20 μl reaction using the High Capacity cDNA Reverse Transcription Kit (Applied Biosystems), according to the kit’s protocol. cDNA was diluted to 2 ng/μl with UV-treated ddH_2_O and stored at −80 °C. Real-time polymerase chain reaction (PCR) was performed using TaqMan Real-Time PCR Assays (Thermo Fisher Scientific) and the QuantStudio 7 Flex Real-Time PCR system (Thermo Fisher Scientific) using two nanograms of cDNA.

### Western blotting

Cells were lysed on ice in fibroblast lysis buffer (25 mM Tris-HCl pH 7.4, 150 mM NaCl, 0.5% Triton-X, 20 mM NH_4_OH) with 1% protease inhibitor cocktail (Set 1; Calbiochem, Merck). An equal amount of protein from each sample was electrophoresed in 8% SDS-PAGE gels and electro-blotted to PVDF membranes (Millipore) as previously described^43^. Bound antibodies were detected using Supersignal West Pico or Femto Chemiluminescent Substate (Thermo Fisher Scientific) and visualised with the ChemiDoc-It Imaging System (UVP, LLC). Relative densitometry was performed using the Fiji software package. Antibodies used for Western blotting were to COL1A1 (NBP1-30054, 1:500, Novus Biologicals), Hsc-70 (sc-7298, 1:2000, Santa Cruz), SMA (A2547, 1:1000, Sigma) and IL-6 (ab6672, 1:500, abcam).

### Single-cell RNA sequencing and analysis

Single-cell RNA sequencing (scRNA-seq) was performed using a custom microfluidic platform^44^, as *per* Macosko *et al*.^25^ with slight adjustments. Filtering and clustering were predominantly carried out using standard functions included in the R Seurat package^45^. Further details are provided in the supporting information.

To filter low-quality events, a number of quality-control (QC) metrics were applied. The majority of these, including the number of unique molecular identifiers (nUMI) and percentage of reads mapping to mitochondrial genes (percent mito) have been described elsewhere previously^1,3^.

In addition, we calculated *per*-droplet estimates of ambient RNA capture. These calculations were based on the assumption that genes most highly expressed across each individual sample are, by probability, those most likely to account for prominent components of ambient RNA. Therefore, low-quality or empty droplets will show enrichment for these genes and a low total number of genes (nGene). To calculate these estimates, we first estimated a lower threshold for nGene, based on the distribution of nGene across all events. This was taken as 2.5 median absolute deviations (MADs) below the median for log_10_(nGene)^46^ (64 genes in this dataset). Using the raw DGE matrix for each sequenced sample, the top 64 highly expressed genes (by total number of reads) were identified across all cells as sample-specific Highly-Expressed Genes (ssHEGs). The percentage of total genes detected (ssHEGs:nGene.genes) or reads (ssHEGs:nGene.counts) per cell composed of these ssHEGs was calculated and used as a relative measure of ambient RNA incorporation.

### Comparing gene expression of *ex vivo* and *in vitro* fibroblasts

*Ex vivo* fibroblasts were filtered by *DCN* expression (identified as a pan-fibroblast marker; cells with a *DCN* expression level of less than 1 were excluded) to exclude any misclassified cells. *In vitro* fibroblasts were expanded (as described in the “Cell Culture” section above) for 1 passage, then cultured in DMEM supplemented with L-glutamine (1% v/v) and FCS (1% v/v) for 5 days, following which they were collected by trypsinisation for Drop-Seq analysis. Cell lines (IMR-90 and H441) were grown to confluence in DMEM supplemented with L-glutamine (1% v/v) and FCS (1% v/v), then collected by trypsinisation for Drop-seq analysis. Cell filtering and clustering was then performed as above on each dataset prior to merging. This resulted in 559 cells for analysis (374 *ex vivo* fibroblasts, 46 *in vitro* NOF, 79 *in vitro* cancer-associated fibroblasts; CAFs, 185 cells from cell lines).

### Statistics

Continuous data with a normal distribution were analysed using the unpaired two-tailed *t*-test. Where normalisation resulted in zero variance in the control sample, *p* values were adjusted using Welch’s correction to account for heteroscedasticity. Comparisons across multiple categories were performed using the ordinary one-way ANOVA. Prism (GraphPad) or R was used to perform statistical analysis and prepare figures. Unless otherwise stated, graphs show mean values +/− the standard error of the mean (S.E.M.). Significance values are denoted as follows: *p* ≥ 0.05: ns, 0.01 ≤ *p* < 0.05: *, 0.001 ≤ *p* < 0.01: **, 0.0001 ≤ *p* < 0.001: ***, *p* < 0.0001: ****. Where appropriate, Bonferroni correction was applied to control for multiple testing.

## Supplementary information

Supplementary Information

## Data Availability

Single-cell RNA sequencing data analysed in this study are available from Gene Expression Omnibus (GSE126111).
