# Supplementary material for: An optimised tissue disaggregation and data processing pipeline for characterising fibroblast phenotypes using single-cell RNA sequencing
Source: Sci Rep. 2019 Jul 3;9:9580. doi: 10.1038/s41598-019-45842-4 (PMC6610623; doi:10.1038/s41598-019-45842-4)
Supplement: Supplementary file 1 — Supplementary Information [file 41598_2019_45842_MOESM1_ESM.pdf]

An optimised tissue disaggregation and data processing pipeline for characterising fibroblast phenotypes using single-cell RNA sequencing

Supplementary Information

Sara Waise[1], Rachel Parker[1], Matthew J.J. Rose-Zerilli[1,2], David M. Layfield[1], Oliver Wood[1], Jonathan West[1,2], Christian H. Ottensmeier[1,3], Gareth J. Thomas[1#], Christopher J. Hanley[1#*]
[1]Cancer Sciences Unit, University of Southampton, UK
[2]Institute for Life Sciences, University of Southampton, UK
[3]Cancer Research UK and NIHR Southampton Experimental Cancer Medicine Centre, UK
[#]Authors contributed equally

Supplementary Methods

Cell culture

In tissue culture substrate coating experiments, 6-well plates were coated with 500µl of either gelatin (0.1% *w/*v; Sigma) or Matrigel® (16 ng/µl; Corning) and incubated for 30 minutes at 37°C, or 1 hour at 4°C, respectively. For three-dimensional cultures, fibroblasts were embedded in a collagen-Matrigel (1:1) gel, prepared as previously described[1]. Confluent primary fibroblasts were re-suspended at $2x10^6$ cells/ml in 500µl of gel and plated to 1 well of a 24-well plate. Gels were incubated at 37°C for 30 minutes to allow setting, before 1ml of DMEM supplemented with L-glutamine (1% v/v; Sigma) and foetal calf serum (FCS; 1% v/v) was added to each well. If not spontaneously detached, gels were detached the following day. Gels were then incubated at 37 °C for 1 week, with the growth medium changed every 72 hours. These gels were then disaggregated by incubation with agitation in Collagenase IV (1 mg/ml; Sigma) at 37 °C for 1 hour. The gel-enzyme mixture was pipetted to disaggregate any remaining fragments, and transferred to an Eppendorf for RNA extraction.

Single-cell RNA sequencing analysis

Single-cell RNA sequencing (scRNA-seq) was performed using a custom microfluidic platform[2]. Drop-seq experiments were performed as *per* Macosko *et al.*[3] with the following adjustments: 15 PCR cycles, 500ng from either 2000 (granulomatous inflammation), 300 (tissue disaggregated for 60 minutes) or 100 (tissue disaggregated for 15 minutes or *in vitro*) cells were used for Nextera XT (Illumina) library preparation. Sample libraries were loaded to a NextSeq 500 sequencer (Illumina) for paired-end read sequencing. Raw sequencing reads were aligned and collapsed onto cell barcodes (corresponding to individual mRNA capture beads) using the Drop-seq toolkit[4]. Digital mRNA transcript counting was performed using the DigitalExpression program[3], creating a digital gene expression (DGE) matrix for downstream analysis.

The DGE was pre-processed to remove low-quality encapsulation events and apoptotic cells as follows: a novel random forest classifier[5] was used to identify low-quality events for removal from further analysis (detailed in the results section). Potentially apoptotic cells were identified as those with between the median and 2.5 MAD above the median values for percent mito, and were excluded from downstream analysis. Likely doublet encapsulation events were identified using a plot showing the total number of genes against the counts *per*

cell: outliers for number of genes (cells with greater than 3000 genes) on this plot were excluded.

Bioinformatic analysis was performed using the Seurat package in R (version 2.3.1, Mac OS)[6]. The filtered DGE matrix (2013 cells, 31587 genes) was transformed by log((counts + 1)/10000), henceforth termed “expression level” for simplicity. Principle component analysis (PCA) dimensionality reduction was performed using the most highly variable genes (identified as outliers on a mean variability plot). Clusters were identified by the Seurat FindClusters function, which uses a shared nearest neighbour-based algorithm. Further dimensionality reduction using t-distributed Stochastic Neighbour Embedding (tSNE) was then carried out to enable visualisation of the clustering results in 2D. Analysis of clustering quality was performed in R using the silhouette analysis function included in the Cluster package (version 2.0.7-1, Mas OS)[7].

Following identification of cell clusters, genes differentially expressed by each cluster were identified using a bimodality test (significance taken as a false discovery rate adjusted $p < 0.001$)[8]. The differentially expressed genes were compared to gene sets from the Immunological Genome[9] and LungGENS[10] projects using the ToppFun gene set enrichment tool[11], to assign a cell type identity to each cluster. The single-cell mRNA-seq data from this publication are available from the Gene Expression Omnibus as GSE126111.

<u>Supplementary Results</u>

Selecting fibroblast markers for FACS analysis

Fibroblasts are known to be heterogeneous with respect to surface marker expression: no single marker will define all fibroblasts. Platelet-derived growth factor alpha (PDGFR-α) has been described as a robust marker of fibroblasts[12], and is expressed by up to 90% of fibroblasts in solid tumours[13]. CD90 (Thy-1) is a glycosylphosphatidyl-inositol-linked cell surface glycoprotein that is expressed by the majority of normal lung fibroblasts[14].

We used FACS to investigate the impact of varying disaggregation enzyme and incubation time on the isolation of different cell types from lung tissues. Immune, epithelial and endothelial cells were identified using the well-described markers CD45[15], EpCAM[16] and CD31[17], respectively. As stated previously, there is currently no single marker that will consistently identify all fibroblasts. Thus, these cells are commonly defined by their lack of expression of markers of other cell lineages. In order to compare accurately the isolation of fibroblasts from tissues, we assessed the suitability of using PDGFR-α, -β and CD90 as positive surface markers of fibroblasts in two primary foetal fibroblast cell lines (skin; HFFF2 and lung; IMR-90; Fig. S1). This showed CD90 to be a highly sensitive marker for IMR-90 cells (99.2% positive, 1175-fold increase in median fluorescence intensity; MFI). CD90 out-performed PDGFR-α and -β (9.2% of cells positive, with a 19-fold increase in MFI), and we therefore used CD90 positivity to identify fibroblasts from tissue samples. It is of note, however, that CD90 was expressed by only 47% of HFFF2 cells: it is not ubiquitously expressed by all cells. It is therefore likely that some fibroblasts will remain unstained using this FACS panel.

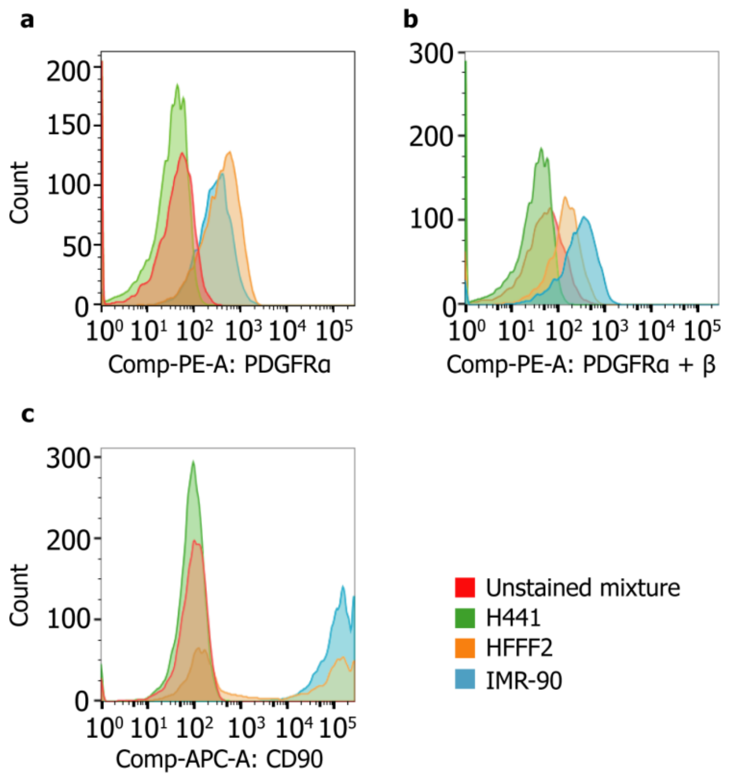

Figure S1. CD90 is a more robust marker of fibroblasts than PDGFRα alone or in combination with PDGFRβ. (**a**-**c**) Histograms showing fluorescence for IMR-90, HFFF2 and H441 cell lines with (**a**) anti-PDGFRα alone, (**b**) anti-PDGFRα and anti-PDGFRβ (**c**) and anti-CD90

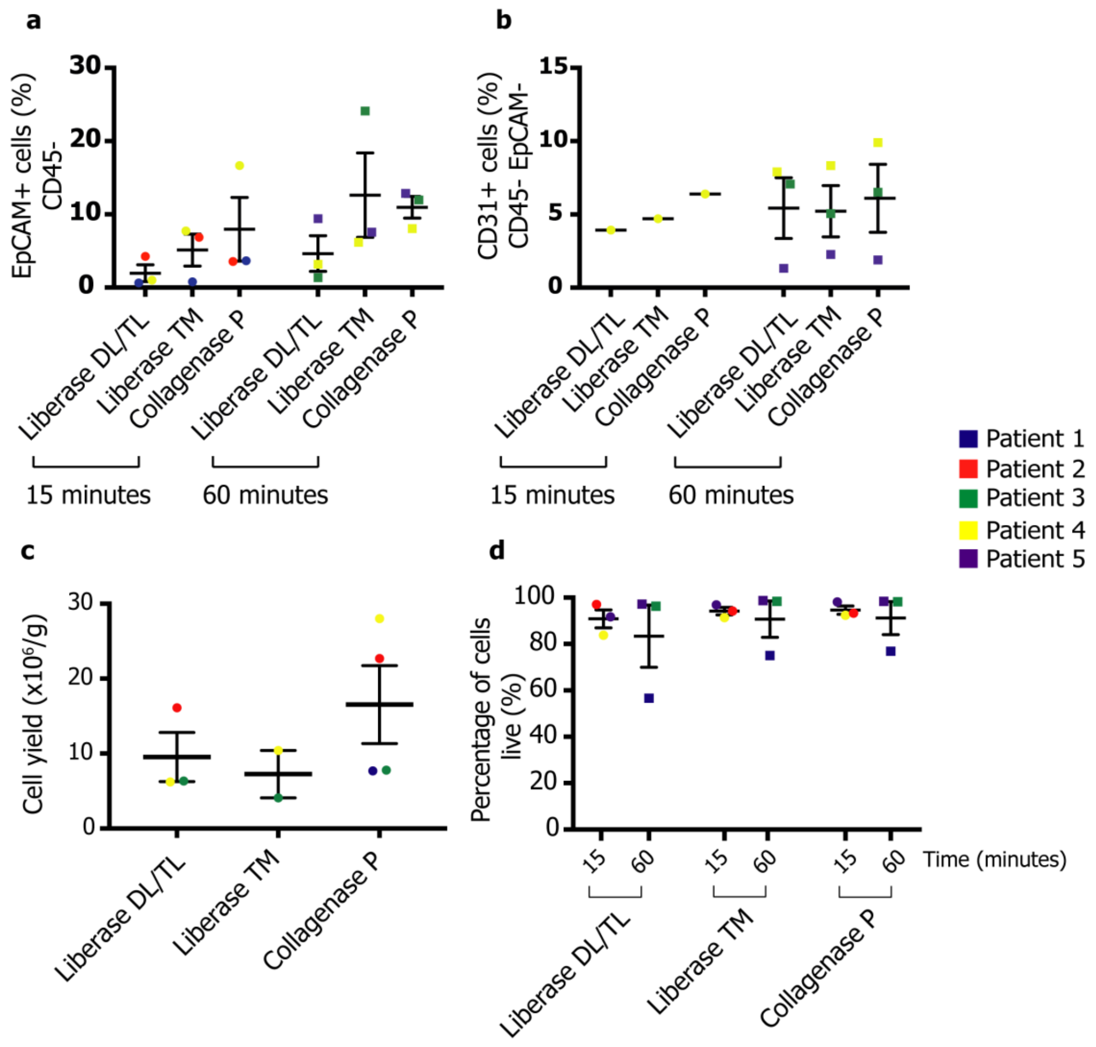

Figure S2. Disaggregation enzyme and duration do not affect the fractions of EpCAM- and CD31-positive cells, cell yield or viability. (**a**) and (**b**) Dot plots showing cell-type fractions isolated by disaggregation procedure across human samples (n=5): Epithelial cells (CD45-EpCAM+: **a**) and endothelial cells (CD45-EpCAM-CD31+; **b**). (**c**) and (**d**) Dot plots showing cell yield (**c**) and cell viability percentage by timepoint (**d**) for different disaggregation procedures across human patient samples (n=5)

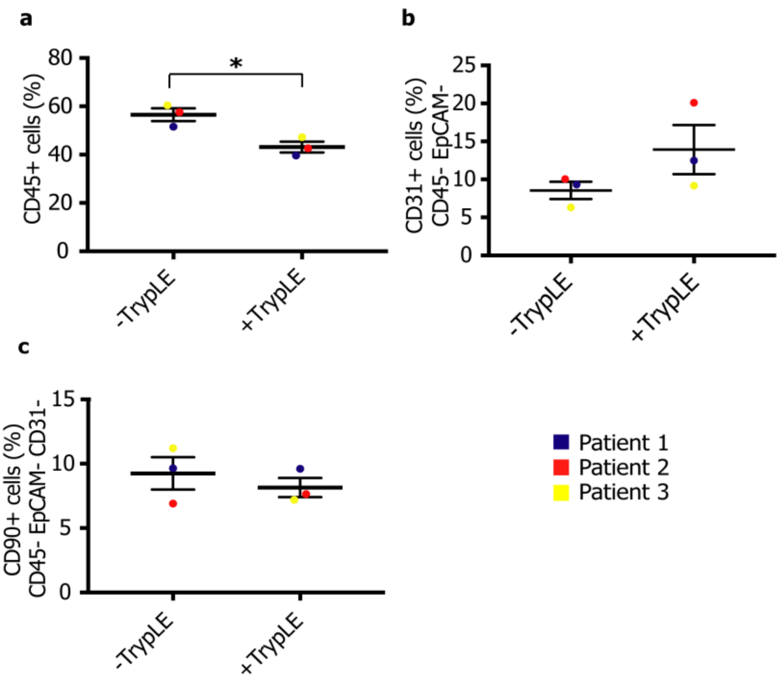

Figure S3. Incubation with TrypLE does not significantly affect the yield of endothelial cells or fibroblasts. (**a**) and (**b**) Dot plots showing cell-type fractions generated by different disaggregation protocols across human samples (n=3): immune cells (CD45+; **a**), endothelial cells (CD45-EpCAM-CD31+; **b**) and fibroblasts (CD45-EpCAM-CD31-CD90+; **c**). **p* < 0.05, unpaired two-tailed *t*-test

Extended Collagenase P disaggregation yields sufficient cDNA for single cell sequencing

Following single-cell transcriptome capture with the Drop-seq platform, a tagmented library molarity in excess of 3 pmol/l is required for subsequent sequencing[3]. Use of the described optimised disaggregation protocol generates in excess of this minimum threshold (Fig. S4), as determined using the BioAnalyzer High Sensitivity Chip (Agilent).

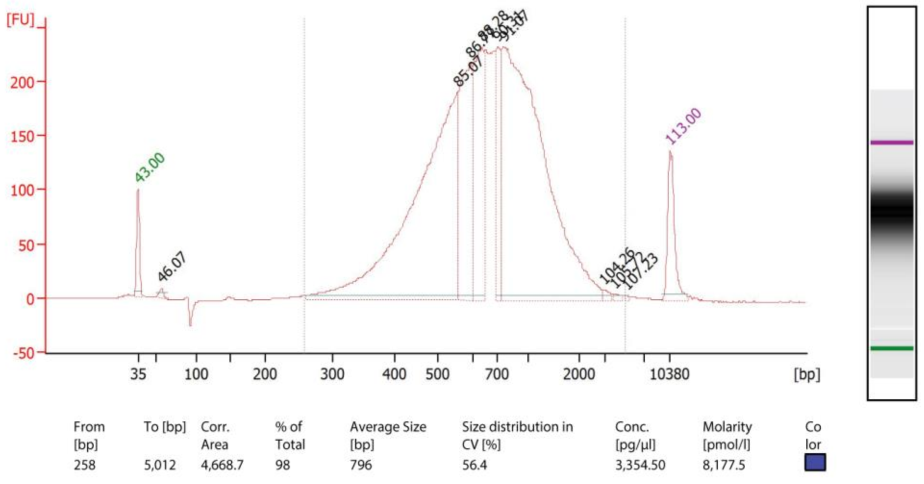

Figure S4. The BioAnalyzer High Sensitivity DNA chip trace

Identifying and removing low-quality droplets

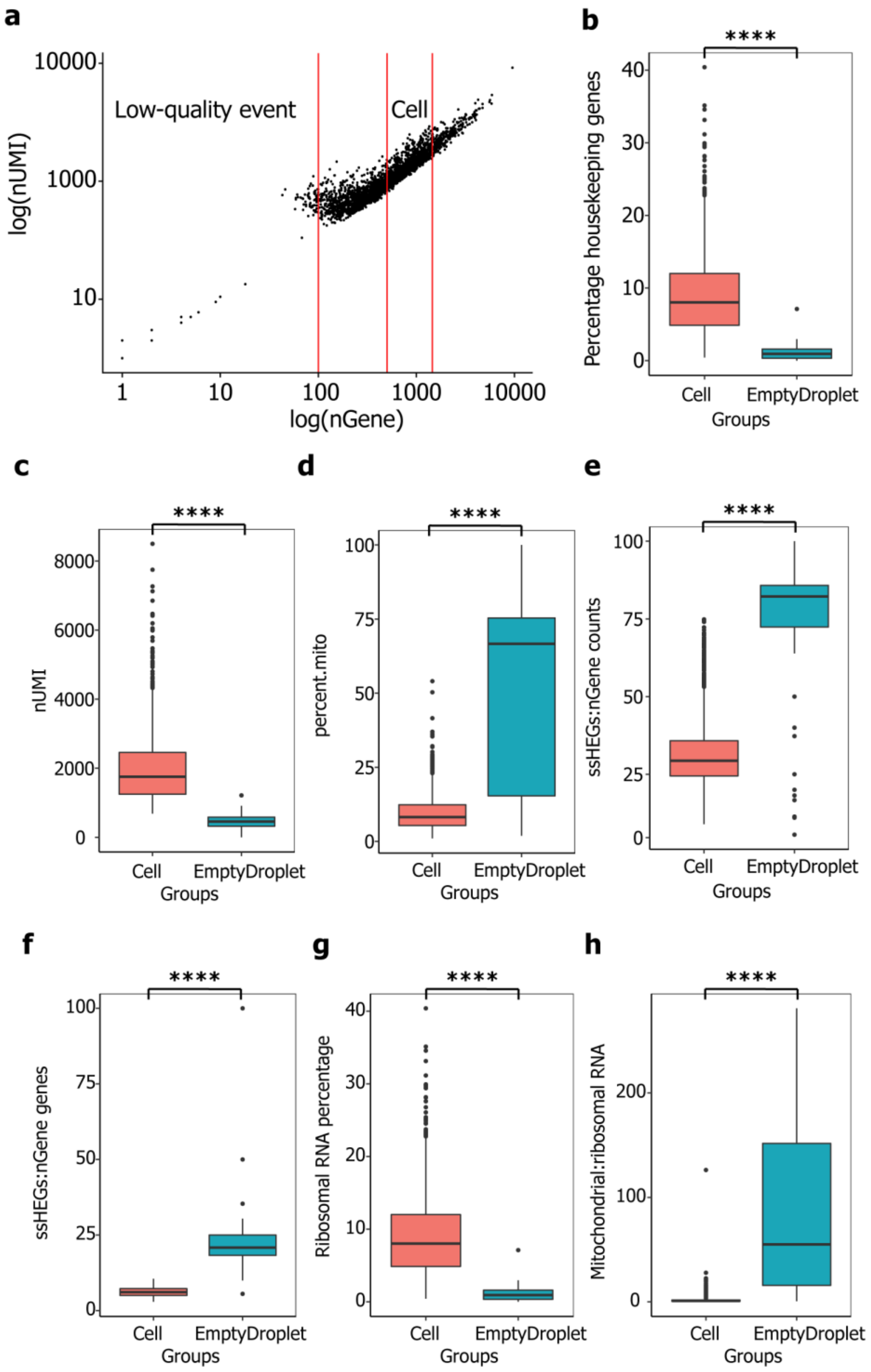

Fig. S5. Identifying quality-control metrics with potential to discriminate between 'low-quality' and 'cell' groups. (**a**) Plot of log(nGene) *vs.* log(nUMI) showing events classified as low-quality or cells. (**b**-**h**) Boxplots showing differences between the low-quality and cell groups for: (**b**): percentage of reads mapping to housekeeping genes, (**c**) nUMI, (**d**) percentage of reads mapping to mitochondrial genes, (**e**) ratio of counts associated with the ambient RNA signature, (**f**) ambient RNA read ratio, (**g**) percentage of reads mapping to ribosomal genes

and (**h**) ratio between percentage of reads mapping to mitochondrial or ribosomal genes. ****$p < 0.0001$, unpaired two-tailed t-test

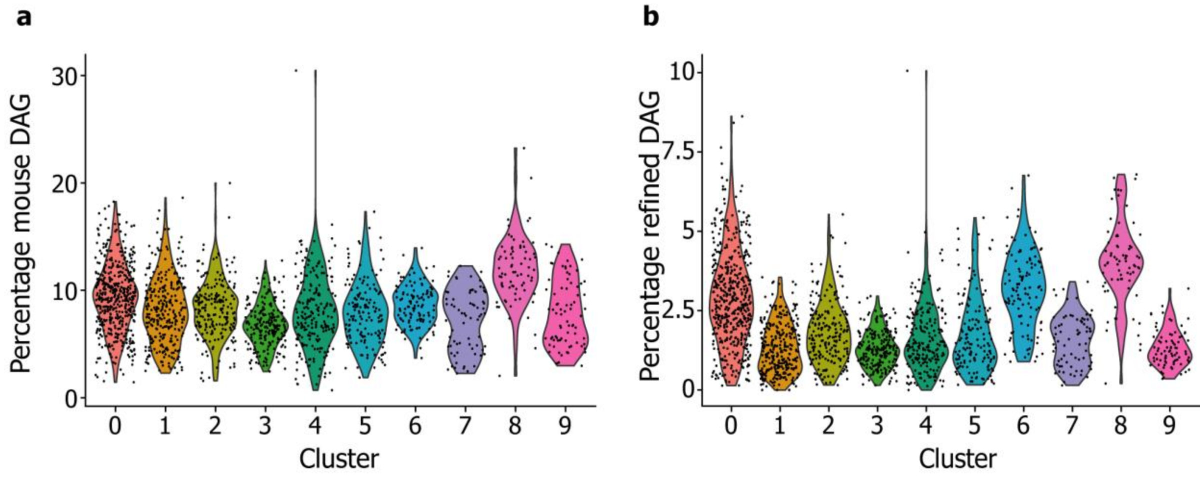

Fig. S6. Expression of the murine and refined disaggregation-associated gene signature is not a prominent feature of any one cluster (cell type). Violin plot showing expression of (**a**) the murine disaggregation-associated signature and (**b**) the refined disaggregation-associated signature across clusters

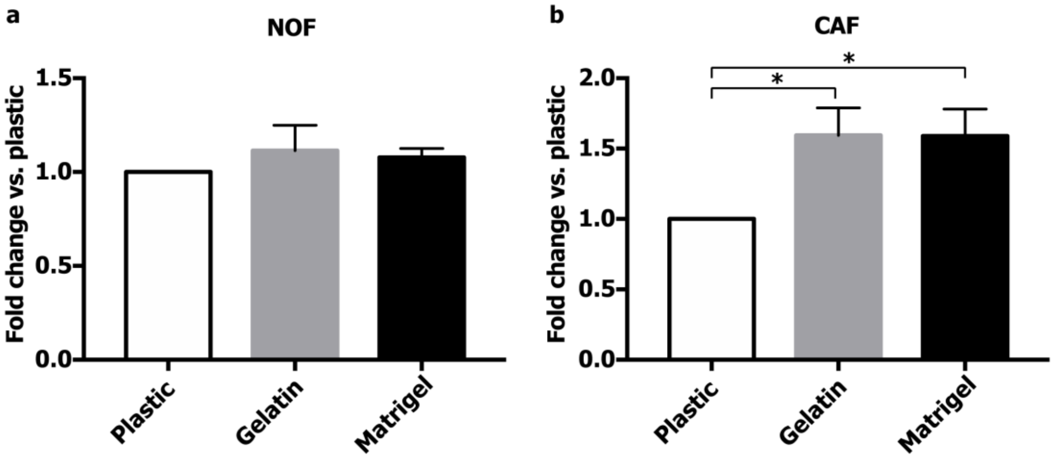

Figure S7. Alteration of culture surface affects proliferation of CAFs. Bar charts showing fold change in fibroblast counts when cultured on plastic, gelatin or Matrigel: (**a**) normal fibroblasts, (**b**) cancer-associated fibroblasts. Counts expressed as fold change relative to plastic (n = 5). *$p < 0.05$, Welch's t-test

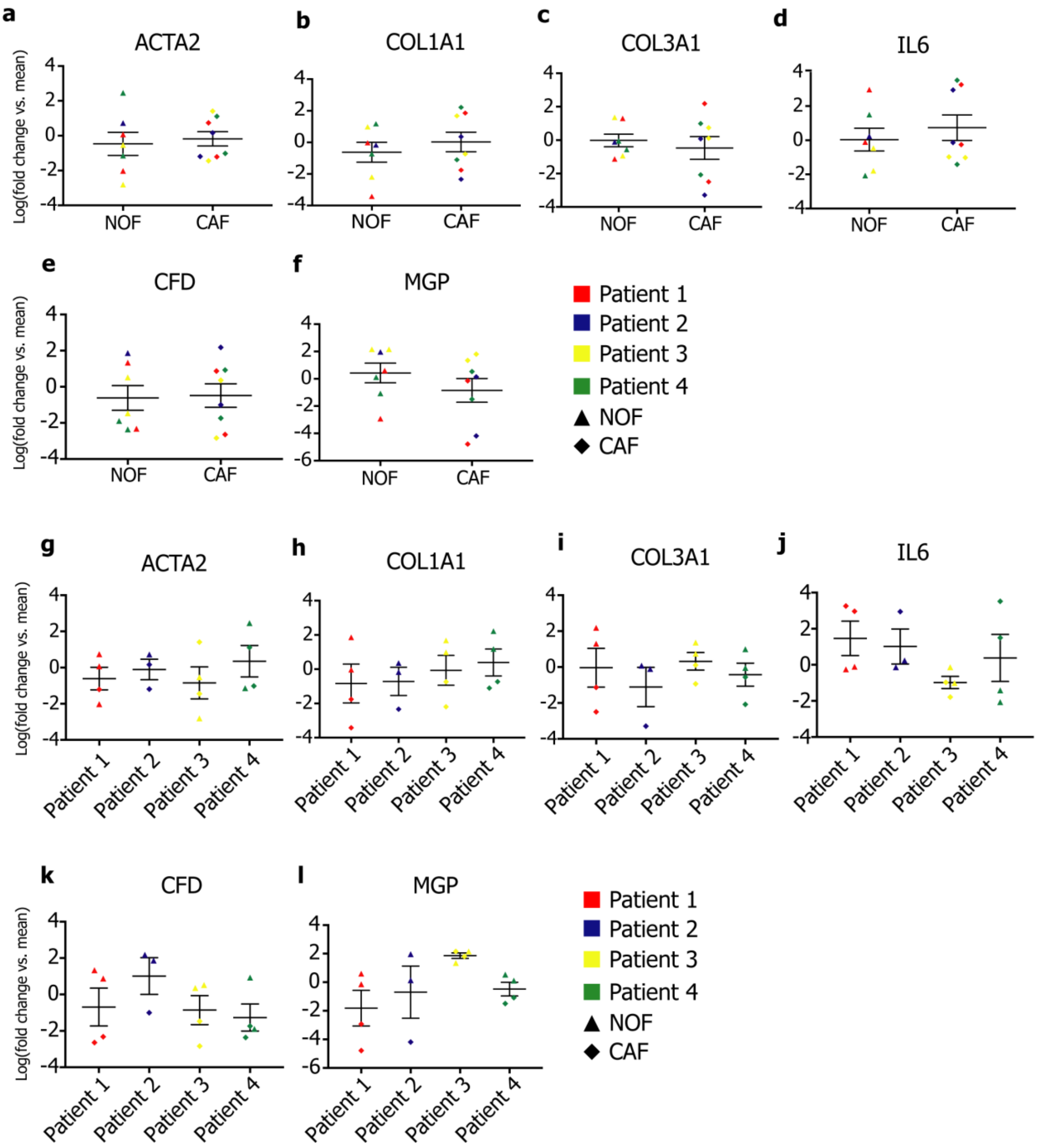

Figure S8. Changes in gene expression across culture substrates are not cell type- or patient-dependent. Dot plots showing changes in gene expression by cell type (**a**-**f**) and across patients (**g**-**l**). Gene expression levels expressed as the log2 fold change relative to mean expression across all samples (n=4). ▲: NOF, ◆: CAF

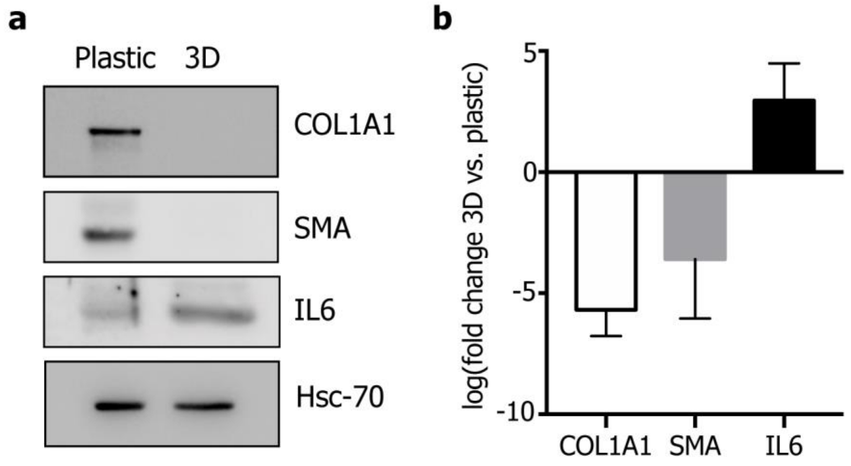

Figure S9. Confirmation of concordance between protein and RNA expression levels for differentially expressed genes. (**a**) Western blot for expression of COL1A1, Hsc-70 (loading control), SMA and IL6. Bar charts showing fold change in measured optical density relative to 2D for (**b**) COL1A1, (**c**) SMA and (**d**) IL6. Graphs show mean values plus the standard deviation for technical replicates (n=3).

1. Nystrom, M. L. *et al.* Development of a quantitative method to analyse tumour cell invasion in organotypic culture. *The Journal of pathology* **205**, 468-475 (2005).
2. Fischlechner, M., West, J. & Zauner, K.-P. DropletKitchen. Github, https://dropletkitchen.github.io/ (2016).
3. Macosko, E. Z. *et al.* Highly Parallel Genome-wide Expression Profiling of Individual Cells Using Nanoliter Droplets. *Cell* **161**, 1202-1214 (2015).
4. McCrow, J. P. Pipeline for automation of Drop-seq data analysis. *Github* http://mccarrolllab.com/dropseq (2016).
5. Paluszyńska, A. Understanding random forests with randomForestExplainer. *The Comprehensive R Archive Network* https://cran.r-project.org/package=randomForestExplainer (2017).
6. Satija, R., Farrell, J. A., Gennert, D., Schier, A. F. & Regev, A. Spatial reconstruction of single-cell gene expression data. *Nature biotechnology* **33**, 495-502 (2015).
7. Maechler, M., Rousseeuw, P., Struyf, A., Hubert, M. & Hornik, K. cluster: Cluster analysis basics and extensions. *Comprehensive R Archive Network* http://cran.r-project.org/package=cluster. (2018).
8. McDavid, A. *et al.* Data exploration, quality control and testing in single-cell qPCR-based gene expression experiments. *Bioinformatics* **29**, 461-467 (2013).
9. Heng, T. S. & Painter, M. W. The Immunological Genome Project: networks of gene expression in immune cells. *Nat Immunol* **9**, 1091-1094 (2008).
10. Du, Y., Guo, M., Whitsett, J. A. & Xu, Y. 'LungGENS': a web-based tool for mapping single-cell gene expression in the developing lung. *Thorax* **70**, 1092-1094 (2015).
11. Chen, J., Bardes, E. E., Aronow, B. J. & Jegga, A. G. ToppGene Suite for gene list enrichment analysis and candidate gene prioritization. *Nucleic Acids Research* **37**, W305-W311 (2009).

12. Erez, N., Truitt, M., Olson, P., Arron, S. T. & Hanahan, D. Cancer-Associated Fibroblasts Are Activated in Incipient Neoplasia to Orchestrate Tumor-Promoting Inflammation in an NF-kappaB-Dependent Manner. *Cancer Cell* **17**, 135-147 (2010).
13. Micke, P. & Ostman, A. Tumour-stroma interaction: cancer-associated fibroblasts as novel targets in anti-cancer therapy? *Lung cancer (Amsterdam, Netherlands)* **45**, S163-175 (2004).
14. Sanders, Y. Y., Kumbla, P. & Hagood, J. S. Enhanced myofibroblastic differentiation and survival in Thy-1(-) lung fibroblasts. *Am J Respir Cell Mol Biol* **36**, 226-235 (2007).
15. Donovan, J. A. & Koretzky, G. A. CD45 and the immune response. *Journal of the American Society of Nephrology : JASN* **4**, 976-985 (1993).
16. Trzpis, M., McLaughlin, P. M. J., de Leij, L. M. F. H. & Harmsen, M. C. Epithelial Cell Adhesion Molecule. *The American Journal of Pathology* **171**, 386-395 (2007).
17. Muller, A. M. *et al.* Expression of the endothelial markers PECAM-1, vWf, and CD34 in vivo and in vitro. *Experimental and molecular pathology* **72**, 221-229 (2002).
